# The Inhibitory Effects and Cytotoxic Activities of the Stem Extract of *Nepenthes miranda* against Single-Stranded DNA-Binding Protein and Oral Carcinoma Cells

**DOI:** 10.3390/plants12112188

**Published:** 2023-05-31

**Authors:** En-Shyh Lin, Yen-Hua Huang, Jo-Chi Chung, Hsin-Hui Su, Cheng-Yang Huang

**Affiliations:** 1 Department of Beauty Science, National Taichung University of Science and Technology, Taichung City 403, Taiwan; 2 Department of Biomedical Sciences, Chung Shan Medical University, Taichung City 402, Taiwan; 3 Department of Pharmacy, Chia Nan University of Pharmacy and Science, Tainan City 717, Taiwan; 4 Department of Medical Research, Chung Shan Medical University Hospital, Taichung City 402, Taiwan

**Keywords:** *Nepenthes miranda*, SSB, anticancer, antipathogen, Ca9-22 gingival carcinoma, GC–MS analysis, *Sinningia bullata*, sitosterol, plumbagin

## Abstract

The carnivorous pitcher plants of the genus *Nepenthes* exhibit many ethnobotanical uses, including treatments of stomachache and fever. In this study, we prepared different extracts from the pitcher, stem, and leaf extracts of *Nepenthes miranda* obtained using 100% methanol and analyzed their inhibitory effects on recombinant single-stranded DNA-binding protein (SSB) from *Klebsiella pneumoniae* (KpSSB). SSB is essential for DNA replication and cell survival and thus an attractive target for potential antipathogen chemotherapy. Different extracts prepared from *Sinningia bullata*, a tuberous member of the flowering plant family Gesneriaceae, were also used to investigate anti-KpSSB properties. Among these extracts, the stem extract of *N. miranda* exhibited the highest anti-KpSSB activity with an IC_50_ value of 15.0 ± 1.8 μg/mL. The cytotoxic effects of the stem extract of *N. miranda* on the survival and apoptosis of the cancer cell lines Ca9-22 gingival carcinoma, CAL27 oral adenosquamous carcinoma, PC-9 pulmonary adenocarcinoma, B16F10 melanoma, and 4T1 mammary carcinoma cells were also demonstrated and compared. Based on collective data, the cytotoxic activities of the stem extract at a concentration of 20 μg/mL followed the order Ca9-22 > CAL27 > PC9 > 4T1 > B16F10 cells. The stem extract of *N. miranda* at a concentration of 40 μg/mL completely inhibited Ca9-22 cell migration and proliferation. In addition, incubation with this extract at a concentration of 20 μg/mL boosted the distribution of the G2 phase from 7.9% to 29.2% in the Ca9-22 cells; in other words, the stem extract might suppress Ca9-22 cell proliferation by inducing G2 cell cycle arrest. Through gas chromatography–mass spectrometry, the 16 most abundant compounds in the stem extract of *N. miranda* were tentatively identified. The 10 most abundant compounds in the stem extract of *N. miranda* were used for docking analysis, and their docking scores were compared. The binding capacity of these compounds was in the order sitosterol > hexadecanoic acid > oleic acid > plumbagin > 2-ethyl-3-methylnaphtho[2,3-b]thiophene-4,9-dione > methyl α-d-galactopyranoside > 3-methoxycatechol > catechol > pyrogallol > hydroxyhydroquinone; thus, sitosterol might exhibit the greatest inhibitory capacity against KpSSB among the selected compounds. Overall, these results may indicate the pharmacological potential of *N. miranda* for further therapeutic applications.

## 1. Introduction

Single-stranded DNA-binding proteins (SSBs) are essential components in DNA metabolic processes, including replication, repair and recombination in both prokaryotes and eukaryotes [1,2]. Single-stranded DNA (ssDNA) is fragile and susceptible, and, thus, it is needed to be protected by SSB from nucleases and chemical attacks. SSB also functions to maintain the transient unwinding of duplex DNA in the single-stranded state in DNA metabolism [3]. Based on these essential roles, SSB is a potential target for drug development.

SSBs bind tightly and cooperatively to ssDNA, regardless of sequence [4]. Bacterial SSBs are typically homotetramers, in which four oligonucleotide/oligosaccharide-binding folds (OB fold) form a DNA-binding domain [5]. SSB also binds to many DNA-binding proteins that constitute the SSB interactome [6]. In eukaryotic cells such as human cells, the equivalent of bacterial SSBs is replication protein A (RPA) [7]. RPA and bacterial SSBs are different in structure, ssDNA-binding mode, and many other functions [8]. For example, the human RPA is active as a heterotrimer composed of three distinctive subunits (RPA1, RPA2, and RPA3) [7]. In addition, RPA interacts with its partner proteins using mechanisms that are distinct from bacterial SSBs. Given the significant structural and functional differences between RPA and bacterial SSBs, the pharmacological inhibition of bacterial SSBs may be used to target pathogens [9].

There is widespread consensus that antimicrobial resistance represents an emerging and alarming threat to human health worldwide [10,11]. Worldwide, growing concern about human and animal infections caused by antibiotic-resistant microorganisms has spurred the interest of the scientific community in antibiotic development [12,13]. As recognized by the Infectious Diseases Society of America [13], ESKAPE pathogens (*Enterococcus faecium*, *Staphylococcus aureus*, *Klebsiella pneumoniae*, *Acinetobacter baumannii*, *Pseudomonas aeruginosa*, and *Enterobacter* species) are of particular concern because they can effectively “escape” the effects of antibacterial drugs [12]. Antibiotic-resistant *K. pneumoniae* are a major cause of hospital- and community-acquired infections, including sepsis, liver abscesses, and pneumonia [14,15]. *K. pneumoniae* are dangerous ESKAPE organisms that are highly correlated with many life-threatening infections and should be suppressed [15]. Recently, we found some extracts of *Nepenthes miranda* capable of suppressing the growth of *K. pneumonia* [16]. Thus, it is worth determining the molecular target(s) in *K. pneumonia* inhibited by extracts of *N. miranda*.

*Nepenthes* are carnivorous plants able to attract and catch small animals such as insects, and retain them in their specialized traps. *Nepenthes* kill and digest these preys to obtain supplemental nutrients for growth, reproduction, and adaptation to nutrient-poor habitats [17]. The genus *Nepenthes* includes almost 120 species [17]. Some extracts of *Nepenthes* plants are used as folk medicines in the treatment of jaundice, hepatitis, gastric ulcers, ureteral stones, diarrhea, diabetes, and high blood pressure. Due to its frequent need for insect attraction and contact, *Nepenthes* may have cytotoxic activities that suppress any contamination by unwanted microbes from insects [18]. These cytotoxic properties may therefore explain why some *Nepenthes* extracts have significant anticancer and antibacterial activities [16,17,18,19,20]. In this study, pitcher, stem, and leaf extracts of *N. miranda* were used to investigate its inhibitory effect on the ssDNA-binding activity of *K. pneumoniae* SSB (KpSSB). *N. miranda* is a new cultivar of a manmade hybrid involving *N. maxima* and *N. northiana* and exhibits unique physiological properties [21]. Prior to this study, whether *Nepenthes* extract can inhibit SSB had remained uninvestigated.

Oral cancer is one of the 10 most common cancers in worldwide, occurring in the oral cavity [22]. Oral squamous cell carcinoma (OSCC) is the most common subsite of head and neck cancer, with a 5-year survival rate of only 50% [23]. The incidence of OSCCs in Asia is more severe depending on the exposure and associated risk factors. Conventional treatments for oral cancers include surgery, chemotherapy, and radiation therapy [24]. However, chemotherapy and radiotherapy cause many adverse effects on normal tissues and cells. In addition, drug resistance is still a severe problem. Therefore, natural compounds are employed as potential anticancer agents, and alternative medicines are also being used for cancer treatment [24]. Accordingly, we examined the cytotoxic effects of *N. miranda* extract on the survival, migration, proliferation, and apoptosis of Ca9-22 gingival carcinoma cells. The cytotoxicity of the *N. miranda* extract against the cancer cell lines CAL27 oral adenosquamous carcinoma, PC-9 pulmonary adenocarcinoma, B16F10 melanoma, and 4T1 mammary carcinoma cells was also demonstrated and compared.

Many plant extracts have been known to help heal human infections due to their secondary metabolite content [25]. DNA replication is one of the most basic biological functions and, thus, SSB, an essential DNA replication protein, should be a prime target in antibiotic development [26]. Recently, we found that the flavonol myricetin can bind to and inhibit *P. aeruginosa* SSB (PaSSB) [27,28,29]. Our complexed crystal structure showed that the binding site of the inhibitor myricetin [29] overlapped with the ssDNA-binding sites of PaSSB [29,30,31,32]. The non-inhibitor quercetin, a myricetin analogue, can also bind to PaSSB; however, quercetin cannot inhibit PaSSB [27]. In this study, we found that myricetin and quercetin cannot inhibit KpSSB. We then screened for possible KpSSB inhibitor(s) from plant extracts. We found that the stem extract of *N. miranda* exhibited significant anti-KpSSB activity. The chemical composition of the stem extracts of *N. miranda* was analyzed via gas chromatography–mass spectrometry (GC–MS). To elucidate the possible inhibition mode, the 10 most abundant compounds in the stem extract of *N. miranda* were used for docking analysis of KpSSB. Further studies should focus on testing these active compounds for the development of drugs against KpSSB and Ca9-22 gingival carcinoma and to determine whether the stem extract of *N. miranda* can be used as an alternative treatment.

## 2. Results

### 2.1. Binding of KpSSB to ssDNA

**Figure 1:**
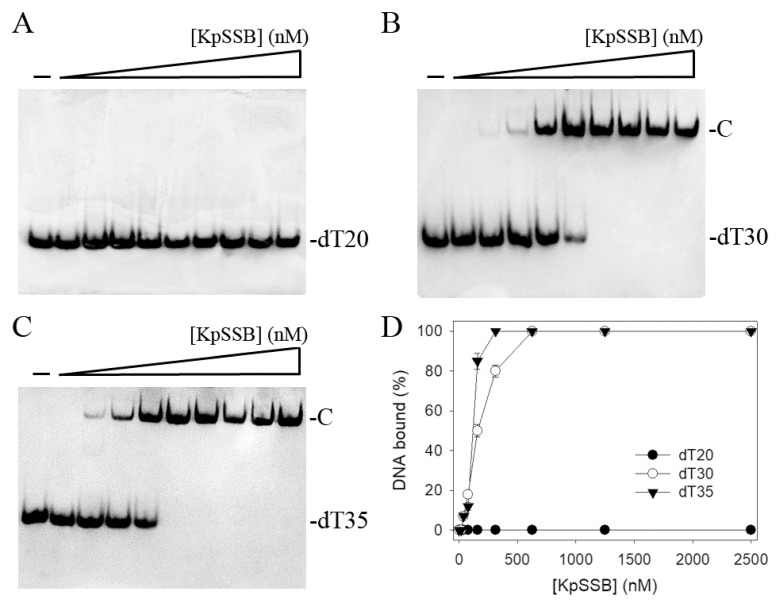
Binding of KpSSB to ssDNA. Purified KpSSB (0, 20, 39, 78, 156, 312, 625, 1250, 2500, and 5000 nM) was incubated with biotin-labeled ssDNA (**A**) dT20, (**B**) dT30, and (**C**) dT35 at 37 °C for 60 min. (**D**) ssDNA-binding abilities of KpSSB. The binding constants ([Protein]_50_) were quantified through linear interpolation based on the protein concentrations. The errors are the standard deviation, as determined over 3 measurements.

Since SSB is required for cell survival, it may be a suitable target for potential antipathogen chemotherapy. Before exploring inhibitor(s) against KpSSB, the binding of KpSSB to ssDNA of different lengths with different protein concentrations was studied using electrophoretic mobility shift analysis (EMSA) [33,34]. The expected result of EMSA for detecting the distinct protein–DNA complex(es) [35] is that when the length of the nucleotides is sufficient for the binding of SSB, the electrophoretic mobility of the SSB–DNA complex will be lower than that of the protein–unbound DNA. Different deoxythymidine (dT) homopolymers were used in this study. These ssDNAs were biotinylated at the 5′ terminal and incubated with purified KpSSB at different concentrations (Figure 1). A streptavidin–horseradish peroxidase conjugate was used to detect these biotin-labeled ssDNAs and their complexes [27,28]. When we incubated KpSSB with a 20-mer dT (dT20), no band shift was observed; that is, KpSSB could not form a stable complex with this homopolymer (Figure 1A). We then tried to use longer ssDNA homopolymers, dT30 (Figure 1B) and dT35 (Figure 1C), for the binding of KpSSB. In contrast to dT20, these longer dT homopolymers produced a very significant band shift (C, complex). These results indicate that KpSSB is capable of forming a stable complex with dT30 and dT35. According to the titration curves of EMSA, the midpoint values for input ssDNA-binding ([Protein]_50_) were quantified. The binding constants of KpSSB to dT30 and dT35 were calculated to be 155 ± 20 and 117 ± 18 nM, respectively (Table 1).

### 2.2. Binding of KpSSB to ssDNA-Containing dsDNA

**Figure 2:**
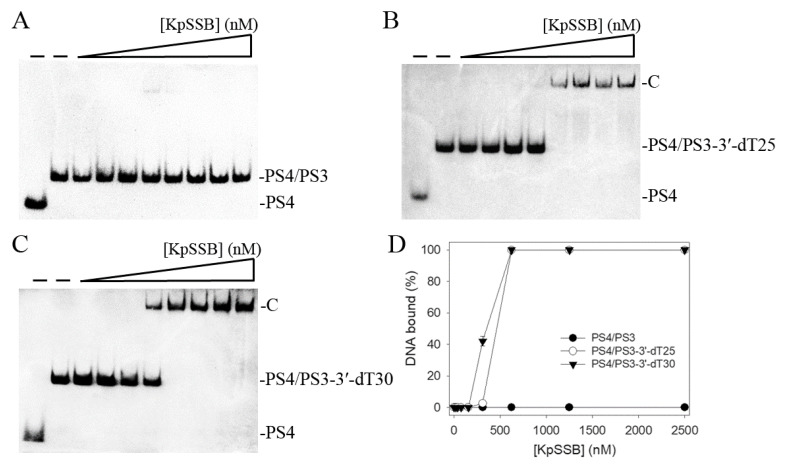
Binding of KpSSB to dsDNA with ssDNA overhang. Purified KpSSB (0, 39, 78, 156, 312, 625, 1250, 2500, and 5000 nM) was incubated with (**A**) PS4/PS3, (**B**) PS4/PS3-3′-dT25, and (**C**) PS4/PS3-3′-dT30 at 37 °C for 60 min. (**D**) DNA-binding abilities of KpSSB. The binding constants ([Protein]_50_) were quantified through linear interpolation based on the protein concentrations. The errors are the standard deviation, as determined over 3 measurements.

We also assessed whether KpSSB can form a complex with double-stranded DNA (dsDNA). The 25-base-pair (bp) dsDNA substrate PS4/PS3 was prepared by annealing two oligonucleotides (PS4 and PS3), of which the DNA strand PS4 was biotinylated. When we incubated KpSSB with PS4/PS3, no band shift was observed. Accordingly, KpSSB could not bind to this dsDNA (Figure 2A). In contrast to PS4/PS3, KpSSB could bind to PS4/PS3 with an ssDNA overhang of 25 and 30 mer dT. The ssDNA overhang was designed at 3′ (PS4/PS3-3′-dT25 and PS4/PS3-3′-dT30). Incubation of PS4/PS3-3′-dT25 (Figure 2B) and PS4/PS3-3′-dT30 (Figure 2C) with KpSSB produced a band shift and formed a stable complex. Through the titration curves of EMSA (Figure 2D), the binding constants of KpSSB to these ssDNA-containing duplex DNAs were calculated and compared. As shown in Table 1, the [Protein]_50_ values of KpSSB for the binding of dT30, PS4/PS3-3′-dT25, and PS4/PS3-3′-dT30 were 155 ± 20, 465 ± 52, and 356 ± 24 nM, respectively. Accordingly, KpSSB preferred binding ssDNA to binding ssDNA-containing dsDNA.

### 2.3. The Flavonols Myricetin, Quercetin, Kaempferol, and Galangin Did Not Inhibit KpSSB

**Figure 3:**
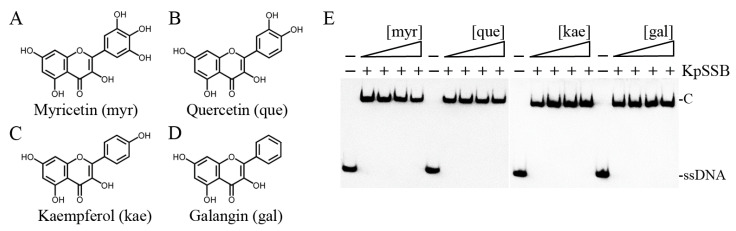
Myricetin is an inhibitor against PaSSB but not against KpSSB. Molecular structure of (**A**) myricetin, (**B**) quercetin, (**C**) kaempferol, and (**D**) galangin. These flavonols were used for inhibiting the activity of KpSSB. (**E**) KpSSB (625 nM) was incubated with each of these flavonols (0, 37.5, 100, and 300 μM). These compounds were dissolved in 10% dimethyl sulfoxide (DMSO). These flavonols did not inhibit the binding of KpSSB to dT30.

Previously, we found that the flavonol myricetin can bind to [29] and inhibit PaSSB [27]. The complexed crystal structure of PaSSB with myricetin revealed where the binding occurs, and we investigated the possible inhibition mode. Residues Lys7, Glu80, Ile105, Asn106, Gly107, and Asn108 in PaSSB were involved in myricetin binding (PDB entry 5YUN), of which Lys7 and Glu80 in PaSSB also interacted with ssDNA (PDB entry 6IRQ) [30,31]. Given that Lys7 and Glu80 are conserved in KpSSB (Lys8 and Glu81; see below), myricetin might also compete with ssDNA for binding sites and inhibit KpSSB. To assess whether myricetin inhibited the binding activity of KpSSB, myricetin (0–300 μM) was included in the binding assay (Figure 3A). A concentration of 625 nM KpSSB, sufficient to reach 100% binding of the dT30 ssDNA (Figure 1), was used for this inhibition test. Unlike the result of PaSSB, myricetin did not inhibit KpSSB binding to dT30. Other myricetin analogues, i.e., quercetin (Figure 3B), kaempferol (Figure 3C), and galangin (Figure 3D) bearing different numbers of hydroxyl substituents on the aromatic rings, were also used for the inhibition test of KpSSB. Each of these flavonols (0–300 μM) was included in the binding assay. We found that these flavonols did not inhibit the binding of KpSSB to dT30 either. Thus, myricetin is an inhibitor against PaSSB but not against KpSSB.

### 2.4. Inhibition of KpSSB by Plant Extracts

**Figure 4:**
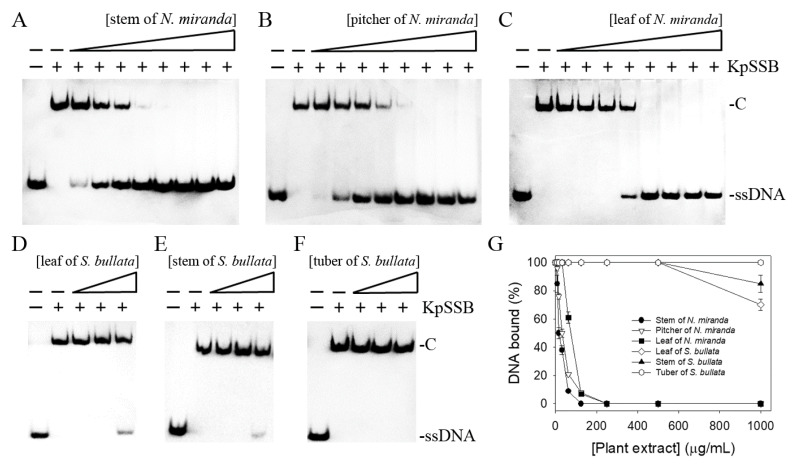
Inhibition of the ssDNA-binding activity of KpSSB by different extracts of *N. miranda* and *S. bullata*. KpSSB (625 nM) was incubated with the methanolic extract of (**A**) the stem of *N. miranda*, (**B**) the pitcher of *N. miranda*, (**C**) the leaf of *N. miranda*, (**D**) the leaf of *S. bullata*, (**E**) the stem of *S. bullata*, and (**F**) the tuber of *S. bullata*. Each of these extracts (at concentrations of 7.8–1000 μg/mL) was included in the binding assay. Among these extracts, the stem of *N. miranda* exhibited the greatest inhibitory effect against KpSSB. (**G**) IC_50_ determinations for KpSSB. The errors are the standard deviation, as determined over 3 measurements.

None of the flavonols used was judged to be an inhibitor against KpSSB (Figure 3). We screened different plant extracts of *N. miranda* and *Sinningia bullata* for a KpSSB inhibitor. *S. bullata* is a tuberous member of the flowering plant family Gesneriaceae [36,37]. Leaf, stem, and pitcher of *N. miranda* and leaf, stem, and tuber of *S. bullata* were extracted using 100% methanol to assess whether these extracts inhibit KpSSB activity (Figure 4). Each of these extracts (at concentrations of 7.8–1000 μg/mL) was included in the binding assay. According to the results from EMSA, the inhibition ability of these extracts was in the following order: the stem extract of *N. miranda* (Figure 4A) > the pitcher extract of *N. miranda* (Figure 4B) > the leaf extract of *N. miranda* (Figure 4C) > the leaf extract of *S. bullata* (Figure 4D) > the stem extract of *S. bullata* (Figure 4E) > the tuber extract of *S. bullata* (Figure 4F). Even at 1000 μg/mL, the tuber extract of *S. bullata* did not influence the binding of KpSSB to ssDNA (Figure 4G). The stem extract of *N. miranda* exhibited the highest inhibition effect against KpSSB among these extracts, with an IC_50_ value of 15.0 ± 1.8 μg/mL (Table 2). Accordingly, certain compounds in the stem extract of *N. miranda* obtained using 100% methanol could be potential KpSSB inhibitors.

### 2.5. Gas Chromatography–Mass Spectrometry (GC–MS) Analysis

**Figure 5:**
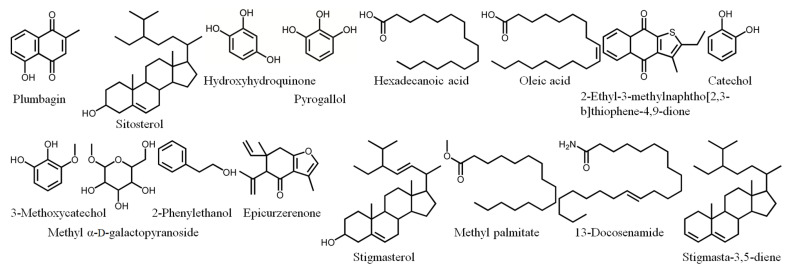
Compounds detected through GC-MS analysis of the stem extract of *N. miranda*. The compounds detected in this extract were tentatively identified by matching generated spectra with NIST 2011 and Wiley 10th edition mass spectral libraries.

Owing to its high inhibitory capability toward KpSSB, the stem extract of *N. miranda* was chosen for GC-MS analysis to determine the compounds present. The compounds detected in this extract were tentatively identified by matching generated spectra with the NIST 2011 and Wiley 10th edition mass spectral libraries. The 16 most abundant compounds (>0.6%) were as follows: plumbagin, sitosterol, hydroxyhydroquinone, pyrogallol, hexadecanoic acid, oleic acid, 2-ethyl-3-methylnaphtho[2,3-b]thiophene-4,9-dione, catechol, 3-methoxycatechol, methyl α-d-galactopyranoside, 2-phenylethanol, epicurzerenone, stigmasterol, methyl palmitate, 13-docosenamide, and stigmasta-3,5-diene (Figure 5).

### 2.6. Dose-Dependent Cytotoxic Effects of the Stem Extract of N. miranda on the Survival of Ca9-22, CAL27, PC9, 4T1, and B16F10 Cells

**Figure 6:**
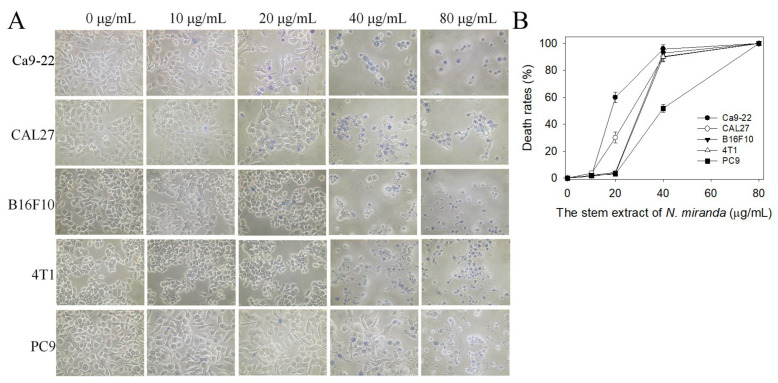
Trypan blue staining assay. (**A**) Cytotoxic effects of the stem extract of *N. miranda* at different concentrations (10, 20, 40, and 80 μg/mL) on the survival of Ca9-22, CAL27, B16F10, 4T1, and PC9 cells. After 0 and 24 h of incubation with the stem extract, trypan blue staining was used to estimate the cell death rate of these cancer cell lines. (**B**) The death rates of the cancer cells treated with the stem extract of *N. miranda*. The cytotoxic activities of 20 μg/mL of the stem extract of *N. miranda* against the cancer cells followed the order Ca9-22 > CAL27 > PC9 > 4T1 > B16F10 cells. The errors are the standard deviation, as determined over 3 measurements.

Given its high inhibitory capability against the SSB, the stem extract of *N. miranda* was also used to test its cytotoxic activities. Initially, a trypan blue staining assay was used to estimate the cytotoxic effects of this extract (Figure 6A). The trypan blue staining assay is based on the principle that live cells possess intact cell membranes that exclude certain dyes, e.g., trypan, whereas dead cells do not [38]. Accordingly, this assay allows for the direct identification and enumeration of live (unstained) and dead (blue) cells in a given population. The cancer cell lines Ca9-22 gingival carcinoma, CAL27 oral adenosquamous carcinoma, PC-9 pulmonary adenocarcinoma, B16F10 melanoma, and 4T1 mammary carcinoma cells were used to evaluate the anticancer activities of the stem extract of *N. miranda*. The monolayers prepared in 96-well microtitration plates for these cancer cells were individually inoculated with this extract at different concentrations (10, 20, 40, and 80 μg/mL) per well. The death rate of cells treated with the stem extract of *N. miranda* was estimated. The cancer cells incubated with 80 μg/mL of this extract were completely killed (Figure 6B). Incubation with 20 μg/mL of the stem extract of *N. miranda* caused the deaths of Ca9-22, CAL27, PC9, 4T1, and B16F10 cells at rates of 60, 30, 5, 4, and 3%, respectively. The cytotoxic activities followed the order Ca9-22 > CAL27 > PC9, 4T1, and B16F10 cells. Thus, the stem extract of *N. miranda* was far more effective against human OSCC cells, i.e., Ca9-22 and CAL27 cells, than against PC9, 4T1, and B16F10 cells.

### 2.7. The Stem Extract of N. miranda Induced Apoptosis of Ca9-22, CAL27, PC9, 4T1, and B16F10 Cells

**Figure 7:**
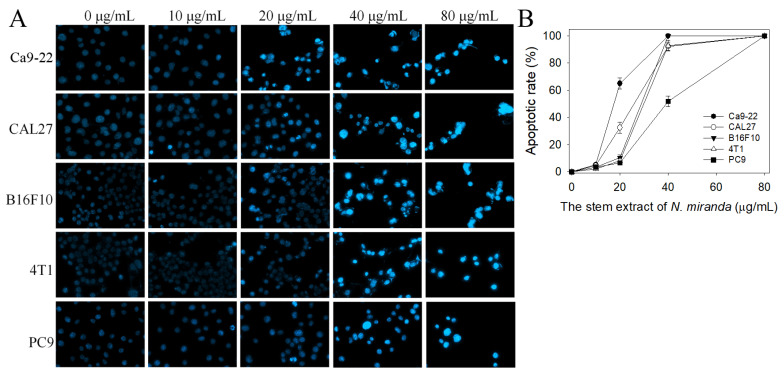
Hoechst staining assay. (**A**) Apoptosis with DNA fragmentation in Ca9-22, CAL27, B16F10, 4T1, and PC9 cells induced by the stem extract of *N. miranda* at different concentrations (10, 20, 40, and 80 μg/mL) was estimated. (**B**) The apoptotic rates of the cancer cells treated with the stem extract of *N. miranda*. The apoptosis-inducing activities of this extract at a concentration of 20 μg/mL followed the order Ca9-22 > CAL27 > PC9 > 4T1 > B16F10 cells. The errors are the standard deviation, as determined over 3 measurements.

Whether the stem extract of *N. miranda* could induce apoptosis of Ca9-22, CAL27, PC9, 4T1, and B16F10 cells was also investigated (Figure 7A). The apoptosis induced by this extract in these cancer cells was estimated by using the Hoechst staining assay. Hoechst 33342 is membrane-permeable and detects all nucleated cells [39]. Through the Hoechst staining assay, we found that the stem extract of *N. miranda*, at a concentration of 80 μg/mL, could induce apoptosis (Figure 7B), with 100% DNA fragmentation identified in these cancer cells. Incubation with 20 μg/mL of the stem extract of *N. miranda* induced apoptosis in Ca9-22, CAL27, PC9, 4T1, and B16F10 cells at the rates of 65, 33, 10, 8, 7%, respectively. Similar to results obtained from the trypan blue staining assay, the apoptosis-inducing activities also followed the order Ca9-22 > CAL27 > PC9, 4T1, and B16F10 cells.

### 2.8. The Stem Extract of N. miranda Exhibited Cytotoxicity against Ca9-22 Cell Migration and Proliferation

**Figure 8:**
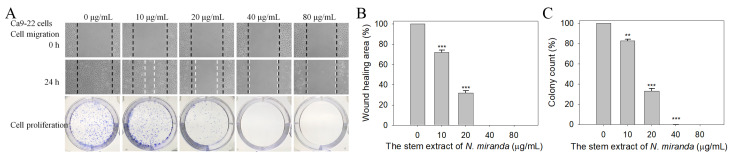
Cytotoxic effects of the stem extract of *N. miranda* on the migration and proliferation of Ca9-22 cells. (**A**) The migration and proliferation of Ca9-22 cells were analyzed using wound-healing and clonogenic formation assays. (**B**) The wound-healing assay. The stem extract of *N. miranda* at a concentration of 40 μg/mL completely inhibited Ca9-22 cell migration. (**C**) A clonogenic formation assay. The stem extract of *N. miranda* at a concentration of 40 μg/mL suppressed the proliferation and colony formation of Ca9-22 cells by 99%. ** *p* < 0.01 and *** *p* < 0.001 compared with the control group.

We found that the stem extract of *N. miranda* also exhibited cytotoxicity against Ca9-22 cell migration and proliferation. The migration and proliferation of Ca9-22 cells were analyzed using wound-healing and clonogenic formation assays, respectively (Figure 8A). Results show that the stem extract of *N. miranda* strongly inhibited the migration and proliferation of Ca9-22 cells. After 24 h of incubation, the stem extract of *N. miranda* at concentrations of 10, 20, 40, and 80 μg/mL reduced Ca9-22 cell migration by 28%, 68%, 100%, and 100%, respectively (Figure 8B). A clonogenic formation assay further revealed that pretreatment with the stem extract of *N. miranda* at concentrations of 10, 20, 40, and 80 μg/mL suppressed the proliferation and colony formation of Ca9-22 cell by 17%, 66%, 99%, and 100%, respectively (Figure 8C). Accordingly, the stem extract of *N. miranda* at a concentration of 40 μg/mL completely inhibited Ca9-22 cell migration and proliferation.

### 2.9. The Stem Extract of N. miranda Suppressed Carcinoma Cell Proliferation by Inducing G2 Cell-Cycle Arrest

**Figure 9:**
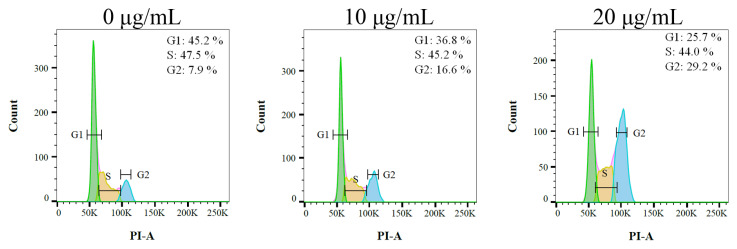
The stem extract of *N. miranda* changed cell cycle progression. Ca9-22 cells were treated with control (0.1% DMSO) and the stem extract of *N. miranda* at the indicated concentrations for 24 h and fixed with 70% alcohol overnight. The cell suspension was stained with propidium iodide (PI) for 30 min and subjected to flow cytometry.

Due to having the highest activities, Ca9-22 cells were chosen for the analysis of the cell cycle progression induced by the stem extract of *N. miranda*. The cell cycle impact was examined using flow cytometry (Figure 9). Incubation with the stem extract of *N. miranda* at concentrations of 10 and 20 μg/mL boosted the distribution of the G2 phase from 7.9% to 16.6% and 29.2% in the Ca9-22 cells, respectively. Therefore, the stem extract of *N. miranda* might suppress carcinoma cell proliferation by inducing G2 cell cycle arrest.

### 2.10. Molecular Docking

**Figure 10:**
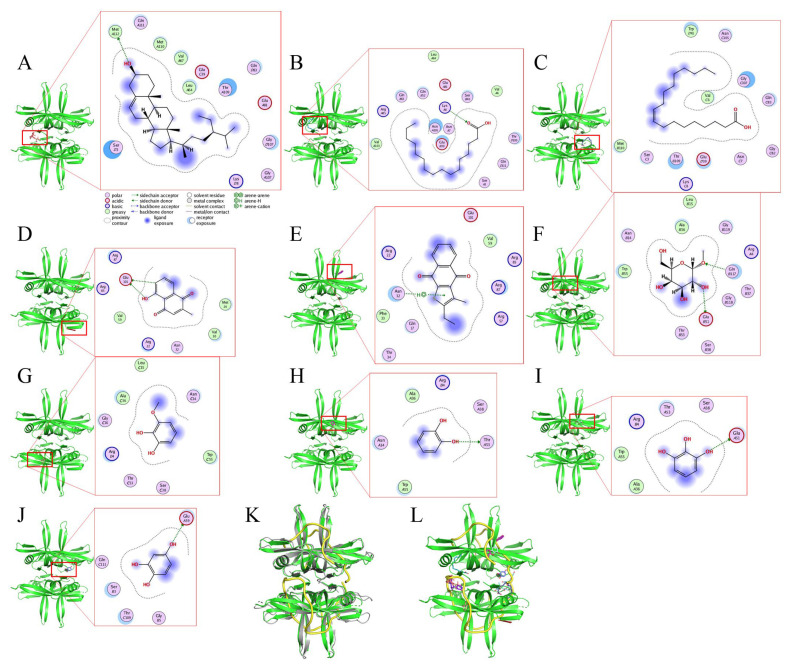
Molecular docking analysis of KpSSB. Interaction analysis is represented by the two-dimensional diagrams, generated using the MOE Dock tool. KpSSB-ligand binding affinities with all possible binding geometries were predicted on the basis of the S score. The binding modes of (**A**) sitosterol (deep salmon), (**B**) hexadecanoic acid (teal), (**C**) oleic acid (deep blue), (**D**) plumbagin (brick red), (**E**) 2-ethyl-3-methylnaphtho[2,3-b]thiophene-4,9-dione (purple), (**F**) methyl α-d-galactopyranoside (sand), (**G**) 3-methoxycatechol (wheat), (**H**) catechol (magenta), (**I**) pyrogallol (palecyan), and (**J**) hydroxyhydroquinone (slate) to KpSSB are shown. (**K**) The superimposed structures of KpSSB and the *Escherichia coli* SSB (EcSSB)–ssDNA complex. The crystal structure reveals that ssDNA (yellow) wraps around EcSSB (gray; PDB ID 1EYG) in a binding topology resembling the seams on a baseball. Given that the structures of KpSSB and EcSSB are similar, the ssDNA mode of KpSSB may resemble that of EcSSB. (**L**) The superimposed structures of the ssDNA and the docked compounds bound by KpSSB. All these compounds seemed likely to occupy ssDNA-binding sites of KpSSB.

Our GC-MS results show that at least 16 compounds were detected in the stem extract of *N. miranda* (Figure 5), and, thus, certain compounds in this extract might be responsible for the inhibition of KpSSB. Recently, we solved the crystal structure of KpSSB [33]; therefore, this structure is available for the study of molecular docking (PDB ID 7F2N). To predict the binding site(s), we elucidated the compound’s mode of binding to KpSSB and calculated the binding energies using the MOE (molecular operating environment) Dock tool (Figure 10). The 10 most abundant compounds in the stem extract of *N. miranda* were used for docking analysis. According to the docking score (the S score), the binding affinities were predicted for the KpSSB–ligand complexes with all possible binding geometries. Based on the S scores (Table 3), the binding capacity of these compounds was in the following order: sitosterol (Figure 10A) > hexadecanoic acid (Figure 10B) > oleic acid (Figure 10C) > plumbagin (Figure 10D) > 2-ethyl-3-methylnaphtho[2,3-b]thiophene-4,9-dione (Figure 10E) > methyl α-d-galactopyranoside (Figure 10F) > 3-methoxycatechol (Figure 10G) > catechol (Figure 10H) > pyrogallol (Figure 10I) > hydroxyhydroquinone (Figure 10J). As it had the highest S score, sitosterol might exhibit the greatest binding affinity toward KpSSB among the selected compounds. According to the modeled structure of the KpSSB–ssDNA complex (Figure 10K), it is likely all these compounds occupy the ssDNA-binding sites of KpSSB (Figure 10L). The KpSSB inhibitory capacity of the stem extract of *N. miranda* might derive from the co-effects of some of these compounds.

## 3. Discussion

Carnivorous plants are polyphyletic and represented by approximately 850 carnivorous plant species [40]. Carnivorous syndromes have evolved independently in approximately 10 lineages of flowering plants [41,42]. Carnivorous plants such as *N. miranda*, which was used in this study, possess specialized traps derived from leaves that are able to attract and catch small animals such as insects and retain them in these pitchers. The captured organisms killed, digested, and assimilated by the plant are a source of additional nutrients for plant growth, reproduction, and adaptation to nutrient-poor habitats. In this study, we found that the stem extract of *N. miranda* is capable of inhibiting the activity of KpSSB (Figure 4) and suppressing the survival, proliferation, and migration abilities of Ca9-22 carcinoma cells (Figure 6 and Figure 8). The stem extract of *N. miranda* also strongly induced apoptosis with DNA fragmentation in the Ca9-22 cells (Figure 7). The flow cytometry results indicate that this extract might suppress Ca9-22 cell proliferation by inducing G2 cell cycle arrest (Figure 9). Given that the G2 cell cycle checkpoint is a critical genome guardian of tumor cells [43] and G2 checkpoint abrogation has been considered to be a promising therapeutic anticancer target [43,44], the cellular signaling pathways that trigger this G2 arrest in Ca9-22 cells caused by the stem extract of *N. miranda* should be further experimentally investigated.

*Nepenthes* has long been used in folk medicine around the world for the treatment of various disorders, such as stomachache and fever [17]. The extracts of *Nepenthes*, due to their (long-time) ethnomedicinal uses, are safe as pharmaceuticals and should have fewer side effects for human use. Thus, more therapeutic applications should be discovered and developed. Recent investigations further confirmed that some *Nepenthes* extracts have significant anticancer and antibacterial activities [16,18,20,45,46]. In this study, we identified SSB as one of the molecular targets inhibited by the *Nepenthes* extracts (Table 2). The stem extract of *N. miranda* exhibited strong anti-KpSSB activity with an IC_50_ value of 15.0 ± 1.8 μg/mL, while extracts of *S. bullata* did not cause a significant effect on the activity of KpSSB. SSB is required for DNA replication, and, thus, the pharmacological inhibition of bacterial SSB may be used to target pathogens. Indeed, suppression of DNA replication and metabolism is widely used as an antimicrobial strategy for antibiotic design. For example, quinolone and aminocoumarin antibiotics, which can be used to target bacterial DNA gyrase and topoisomerase IV, were successfully developed for clinical applications [47,48]. Thus, it is worth continuing the search for inhibitors against SSB.

Many valuable metabolites have been identified in different *Nepenthes* species [19]. It is assumed that the majority of the biological activities of *Nepenthes* species are related to naphthoquinones. For example, plumbagin derived from *N. alata* showed significant anticancer activity [20]. In animal models, plumbagin-treated mice showed a significant reduction in tumor growth, and no side effects [49]. In this study, the most abundant compound in the stem extract of *N. miranda* analyzed via GC-MS was plumbagin (12.9%), and, thus, the cytotoxic properties of this extract against Ca9-22 cells may be related to plumbagin. In addition, sitosterol, a phytosterol possessing significant anticancer activities against a variety of cancers [50,51], was the second most abundant compound (6.5%) found in the stem extract of *N. miranda*. Accordingly, the stem extract of *N. miranda* might induce apoptosis and cause cancer cell death mainly via the co-activities of plumbagin and sitosterol. The stem extract of *N. miranda* used was much more efficient against OSCC cells, i.e., Ca9-22 and CAL27 cells, than against PC9, 4T1, and B16F10 cells. Thus, it is of considerable interest to further explain why the cytotoxic specificity of this extract differs for possible medical applications. Given that OSCC is the most common subsite of head and neck cancer with a 5-year survival rate of only 50% [23], it is worth determining whether *N. miranda* extracts could be a potential natural alternative medication or complementary therapy, e.g., though its use as an adjuvant or ointment for the treatment of oral cancer.

Like the stem extract of *N. miranda*, the leaf extract of *S. bullata* also exhibited cytotoxicity in regard to cancer cell survival, migration, and proliferation, and induced apoptosis in cancer cells [37]. However, their contents, as tentatively identified via GC-MS, were found to be significantly different. Thus, the cytotoxic mechanisms may differ between these two extracts. Plumbagin, a major active compound in the stem extract of *N. miranda*, was not found in the leaf extract of *S. bullata* [37], but other naphthoquinones such as 7,8-dimethoxydunnione [52] and aggregatins [53] were found in the tuber extracts of similar *Sinningia* species. These and other naphthoquinones could possibly be useful in combination for further anticancer drug development.

**Figure 11:**
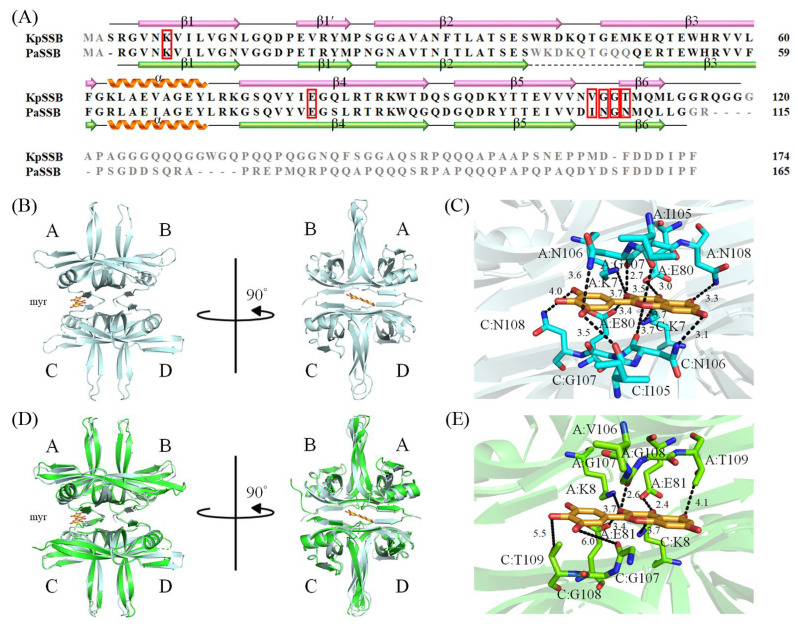
Structural analysis of KpSSB. (**A**) Sequence alignment of KpSSB and PaSSB. The secondary structural elements of KpSSB and PaSSB are shown with the sequences. Residues not observed in the crystal structure of KpSSB (PDB ID 7F2N) and PaSSB (PDB ID 5YUN) are in gray. Residues K7, E80, I105, N106, G107, and N108 in PaSSB are involved in myricetin binding (boxed in red). The corresponding residues in KpSSB are K8, E81, V106, G107, G108, and T109. Only K8 and E81 in KpSSB were conserved as possible sites for myricetin binding. (**B**) The complexed crystal structure of PaSSB. Myricetin sandwiched by PaSSB monomers A and C is in bright orange. (**C**) The myricetin-binding mode to PsSSB. (**D**) The superimposed structures of KpSSB and the myricetin-complexed PaSSB. (**E**) The proposed myricetin-binding mode of KpSSB. Several interactions in the myricetin–PaSSB complex were not found in this modeled myricetin–KpSSB complex. This may explain why myricetin cannot inhibit KpSSB because the myricetin-interacting residues are significantly different in KpSSB.

The development of clinically useful small-molecule antibiotics has been a seminal event in the field of infectious diseases [10,12]. In this study, we found that the stem extract of *N. miranda* can strongly inhibit the ssDNA-binding activity of KpSSB (Figure 4). The 10 most abundant compounds in this extract, which are possibly responsible for the inhibition of KpSSB, were used for docking analysis (Figure 10). The compounds in the stem extract of *N. miranda* could occupy the ssDNA-binding sites of KpSSB; therefore, the KpSSB inhibitory capacity might derive from the co-effects of some of these compounds. SSB is essential for all aspects of DNA metabolism, such as DNA replication, repair, recombination, and replication restart [1,6,54], and thus potentially represents an attractive target in antipathogen chemotherapy. Previously, we found that the flavonol myricetin can bind to and inhibit PaSSB [27,29]. The binding site of the inhibitor myricetin was found to overlap with the ssDNA-binding sites in PaSSB [30,31]. Given that the crystal structure of KpSSB is similar to that of PaSSB [32,33], one might conclude that the PaSSB inhibitor myricetin must inhibit KpSSB. However, myricetin, as well as quercetin, kaempferol, and galangin, did not inhibit the ssDNA-binding activity of KpSSB (Figure 3). Both residues K7 and E80, crucial myricetin and ssDNA-binding sites in PaSSB (Figure 11A), are conserved in KpSSB (K8 and E81). However, other myricetin-interacting residues in PaSSB, namely I105, N106, G107, and N108, are not conserved in KpSSB (Figure 11B,C). Correspondingly, these residues in KpSSB are V106, G107, G108, and T109 (Figure 11A). This may explain why myricetin cannot inhibit KpSSB because the myricetin-binding residues are significantly different in KpSSB. For example, superposition analysis (Figure 11D) indicated that the myricetin-interacting distances of residues G107 and T109 in KpSSB (subunit C) were 6.0 and 5.5 Å, respectively (Figure 11E). Based on these distances (>5 Å), these two residues in KpSSB may play no role in myricetin binding. However, this speculation must be further biochemically and structurally investigated.

In conclusion, this study was the first to identify the anti-KpSSB capacity of the stem extract of *N. miranda*. The cytotoxic effects of this extract on the survival, apoptosis, proliferation, and migration of Ca9-22 cells were also examined. The most abundant compounds in this extract were determined via GC–MS to obtain a better understanding of which compounds may be active, alone, or acting in combination in these biological activities. These results may indicate the pharmacological potential of the stem extract of *N. miranda* for possible medical applications.

## 4. Materials and Methods

### 4.1. Chemicals, Cell Lines, and Bacterial Strains

The chemicals myricetin, quercetin, kaempferol, and galangin were obtained from Sigma-Aldrich (St. Louis, MO, USA). Restriction enzymes and DNA-modifying enzymes were purchased from New England Biolabs (Ipswich, MA, USA). The *E. coli* strains TOP10F’ (Invitrogen, Waltham, MA, USA) and BL21(DE3) pLysS (Novagen, Worcestershire, UK) were used for genetic construction and protein expression, respectively. The cell lines Ca9-22 gingival carcinoma, CAL27 oral adenosquamous carcinoma, PC-9 pulmonary adenocarcinoma, B16F10 melanoma, and 4T1 mammary carcinoma were obtained from Food Industry Research and Development Institute, Hsinchu, Taiwan [16,18,37,45,55,56]. Ca9-22, CAL27, B16F10 and 4T1 cells were maintained as a monolayer culture in Dulbecco’s modified Eagle medium (GibcoTM; Thermo fisher Scientific, Waltham, MA, USA) supplemented with 10% fetal bovine serum (FBS), 100 unit/mL penicillin, and 100 μg/mL streptomycin. PC-9 cells were maintained in RPMI 1640 with 10% FBS, 100 unit/mL penicillin, and 100 μg/mL streptomycin. Cells were incubated at 37 °C in a 95% air and 5% CO_2_ incubator.

### 4.2. Recombinant Protein Expression and Purification

Briefly, the KpSSB expression plasmid pET21b-KpSSB [57,58,59] was transformed into *E. coli* BL21 (DE3) cells. The resultant cells were grown to OD_600_ of 0.9 in LB medium containing 250 μg/mL ampicillin with rapid shaking at 37 °C. The overexpression was induced by incubating with 1 mM isopropyl thiogalactopyranoside for 9 h at 25 °C. The cells overexpressing the protein were chilled on ice, harvested by centrifugation, resuspended in Buffer A (20 mM Tris–HCl, 5 mM imidazole and 0.5 M NaCl, pH 7.9) and disrupted by sonication with ice cooling. The recombinant protein was purified from the soluble supernatant using Ni^2+^-affinity chromatography (HisTrap HP; GE Healthcare Bio-Sciences, Piscataway, NJ, USA), eluted with Buffer B (20 mM Tris-HCl, 250 mM imidazole, and 0.5 M NaCl, pH 7.9), and dialyzed against a dialysis buffer (20 mM HEPES and 100 mM NaCl, pH 7.0; Buffer C). Protein purity remained at >97% as determined by SDS-PAGE (Mini-PROTEAN Tetra System; Bio-Rad, Hercules, CA, USA).

### 4.3. Preparation of dsDNA Substrates

The dsDNA substrate PS4/PS3 was prepared with a biotinylated PS4 strand (3′-GGGCTTAAGCCTATCGAGCCATGGG-5′; 25 mer) and an unlabeled PS3 strand (5′-CCCGAATTCGGATAGCTCGGTACCC-3′; 25 mer) [60]. The ssDNA strands were used at a 1:1 concentration ratio to prepare each dsDNA substrate used in this study. dsDNA substrates PS3-3′-dT25 and PS3-3′-dT30 were prepared with a biotinylated PS4 and an unlabeled PS3-3′-dT25 (5′-CCCGAATTCGGATAGCTCGGTACCC-dT25-3′) or PS3-3′-dT30 strand. Each dsDNA substrate, in 20 mM HEPES (pH 7.0) and 100 mM NaCl, was formed through brief heating at 95 °C for 5 min followed by slow cooling to room temperature overnight.

### 4.4. EMSA

dT20, dT30, and dT35 were biotinylated at the 5′ terminal and incubated with purified KpSSB of different concentrations (0, 20, 39, 78, 156, 312, 625, 1250, 2500, and 5000 nM). Including dsDNA substrates PS4/PS3, PS4/PS3-3′-dT25, and PS4/PS3-3′-dT30, the final concentration of these DNA substrates for analysis was 30 fmol/μL. EMSA was performed using the LightShift Chemiluminescent EMSA Kit (Thermo Scientific, Waltham, MA, USA) [27,28]. In brief, KpSSB was incubated for 60 min at 37 °C with DNA substrate in a total volume of 6 μL in 40 mM Tris–HCl (pH 7.5) and 50 mM NaCl. Following incubation, 4 μL of a dye mixture (0.01% bromophenol blue and 40% glycerol) was added. The resulting samples were loaded and resolved on native polyacrylamide gel (8%) at 100 V for 1 h in TBE running buffer (89 mM Tris borate and 1 mM EDTA). The protein–DNA complexes were electroblotted to positively charged nylon membrane (GE, Boston, MA, USA) at 100 V for 30 min in TBE buffer. Transferred DNA was cross-linked with nylon membrane using a UV-light cross-linker instrument equipped with 312 nm bulbs for a 10 min exposure. Using streptavidin–horseradish peroxidase conjugate and chemiluminescent substrate contained in SuperSignal™ West Atto Ultimate Sensitivity Substrate (Pierce Biotechnology, Waltham, MA, USA), biotin-labeled DNA was detected. The DNA-binding ability of KpSSB ([Protein]_50_) was estimated through linear interpolation from the concentration of the protein that bound 50% of the input DNA.

### 4.5. Inhibition Assay

EMSA for an inhibition test against KpSSB was conducted using the protocol described previously [27,28]. A biotinylated dT30 was used as substrate for this inhibition assay. KpSSB (625 nM) was incubated with dT30 and the indicated compound (0, 37.5, 100, and 300 μM) for 60 min at 37 °C. Indicated plant extracts (7.8–1000 μg/mL) were also included in the binding assay for an inhibition test against KpSSB. Following incubation, the resultant KpSSB solution was analyzed using EMSA. The titration curves were generated and the concentration of the compound or plant extract required for 50% inhibition (IC_50_) was determined directly from the graphic analysis.

### 4.6. Plant Materials and Extract Preparations

Briefly, *N. miranda* (leaf, stem, and pitcher) [16] and *S. bullata* (leaf, stem, and tuber) [37], obtained at Guoguang Flower Market and Taiwan Provincial Flower Marketing Cooperative and identified by Dr. Zhong-Bao Zhang in December 2020, were collected, dried, cut into small pieces, and pulverized into powder. Extractions were carried out by placing 1 g of plant powder into 250 mL conical flask. A total of 100 mL of methanol was added to the flask and shaken on an orbital shaker for 5 h. The resultant extract was filtered using a 0.45 μm filter. Solvent (methanol) in extracts was removed via a hot air circulation oven at 40 °C. Extracts were stored at −80 °C until use. The extract powder was dissolved in 20% DMSO to make a stock solution at a concentration of 20 mg/mL. For the anticancer cell assays, the stock was diluted with the supplemented culture medium to the indicated assay concentrations. The cancer cells were incubated with the resultant extract solutions or the culture medium with 0.2% DMSO, reference as treatment, or the control group. For EMSA, the stock was diluted with Buffer B to the indicated assay concentrations. The protein was incubated with the resultant extract solutions or Buffer B with 1% DMSO, reference as treatment, or the control group.

### 4.7. GC-MS Analysis

The molecular composition of samples was determined using GC-MS analysis. The Thermo Scientific TRACE 1300 Gas Chromatograph with a Thermo Scientific ISQ Single Quadrupole Mass Spectrometer System was used to analyze the filtered sample. A Rxi-5ms column (30 m × 0.25 mm i.d. × 0.25 μm film) was used. Helium was used as the carrier gas at a constant flow rate of 1 mL/min. The initial oven temperature was 40 °C. It was maintained at 40 °C for 3 min, and then the temperature was gradually increased to 300 °C at a rate of 10 °C/min. This temperature was maintained for 1 min. The temperature of the injection port was 300 °C and the flow rate of helium was 1 mL/min. The compounds discharged from the column were detected using a quadrupole mass detector. The ions were generated by electron ionization method. The temperatures of the MS quadrupole and source were 150 and 300 °C, respectively, the electron energy was 70 eV, the temperature of the detector was 300 °C, the emission current multiplier voltage was 1624 V, the interface temperature was 300 °C, and the mass range was from 29 to 650 amu. The peak area normalization method was used to determine the relative mass fraction of each chemical component. Compounds were identified by matching generated spectra with NIST 2011 and Wiley 10th edition mass spectral libraries.

### 4.8. Trypan Blue Cytotoxicity Assay

The trypan blue cytotoxicity assay was performed to assess cell death [38]. Briefly, Ca9-22, CAL27, B16F10, PC-9, and 4T1 cells (1 × 10^4^) were incubated with the extract of *N. miranda* in a 100 μL volume. After 24 h, a trypan blue staining assay was used to estimate the cytotoxic activity exhibited by the extract.

### 4.9. Chromatin Condensation Assay

The apoptosis in cancer cells was assayed with Hoechst 33342 staining [39]. Briefly, Ca9-22, CAL27, B16F10, PC-9, and 4T1 cells were seeded in 96-well plates at a density of 5 × 10^3^ cells per well. Culture medium was 200 μL per well. Cells were allowed to adhere for 16 h. Cells were incubated with the extract of *N. miranda* for 24 h, washed with PBS, and stained with the Hoechst dye (1 μg/mL) in the dark at RT for 10 min. ImageXpress Pico (Molecular Devices, Silicon Valley, CA, USA) was used to image cells. Image acquisition was performed on each well using a 20× magnification, 6 × 6 square image scan, on the DAPI filter cubes. Image analyses were performed using the CellReporterXpress Version 2 software. The apoptotic index was calculated as follows: apoptotic index = apoptotic cell number/(apoptotic cell number + nonapoptotic cell number).

### 4.10. Clonogenic Formation Assay

To assess the inhibition of Ca9-22 cell proliferation, a clonogenic formation assay [61] was used. Briefly, Ca9-22 cells were seeded at a density of 1 × 10^3^ cells per well into 6-well plates and incubated overnight for attachment. The cells were incubated with the extract of *N. miranda* for 5–7 days. After washing with PBS, formed colonies were fixed with methanol and stained with 0.5% crystal violet for 20 min. The number of colonies was counted under a light microscope.

### 4.11. Wound-Healing Assay

To analyze the inhibition of Ca9-22 cell migration, the wound-healing assay [62] was performed. Briefly, Ca9-22 cells were seeded in 96-well plates and incubated in serum-reduced medium for 6 h. The cells were then wounded in a line across the well with a 200 μL pipette tip and washed twice with the serum-reduced medium. The cells were treated with the extract of *N. miranda* and incubated for 24 h to allow migration.

### 4.12. MOE Dock Analysis

MOE Dock [63] was used to analyze binding of sitosterol, hexadecanoic acid, oleic acid, plumbagin, 2-ethyl-3-methylnaphtho[2,3-b]thiophene-4,9-dione, methyl α-d-galactopyranoside, 3-methoxycatechol, catechol, pyrogallol, and hydroxyhydroquinone to KpSSB. Their binding capacity in KpSSB was also calculated for comparison. Initially, the water molecules in the crystal structure of KpSSB (PDB ID 7F2N) [33] were removed via MOE. Through 3D protonation with subsequent minimization of energy, hydrogen atoms were added to the protein structure. Top-ranked confirmations were developed and analyzed.

### 4.13. Flow Analysis

Flow cytometry was used for cell cycle analysis. Ca9-22 cells were treated with DMSO or the extract of *N. miranda* for 24 h and harvested with trypsin. The harvested cells were washed, resuspended in PBS with 1% FBS, and then fixed with cold ethanol (70%). Fixed cells were washed and incubated in PBS buffer for 5 min. The resultant cells were resuspended in PI/RNase solution (PBS, RNase, and 50 μg/mL PI) and stained for 30 min at 37 °C in the dark. A BD FACSCanto II (BD Biosciences, San Jose, CA, USA) was used to analyze these treated cells. Through FlowJo v10 software (Tree Star, Inc., Ashland, OR, USA), the distribution of each phase was calculated and visualized.

## Figures and Tables

**Table 1:** ssDNA-binding properties of KpSSB, as analyzed using EMSA.

DNA	[Protein]_50_ (nM)
dT20	ND
dT30	155 ± 20
dT35	117 ± 18
PS4/PS3	ND
PS4/PS3-3′-dT25	465 ± 52
PS4/PS3-3′-dT30	356 ± 24

Values show mean standard deviation of at least three independent experiments.

**Table 2:** Inhibition of the ssDNA-binding activity of KpSSB.

Inhibitor	IC_50_
Myricetin	N.D.
Quercetin	N.D.
Kaempferol	N.D.
Galangin	N.D.
Stem extract of *Nepenthes miranda*	15.0 ± 1.8 μg/mL
Pitcher extract of *Nepenthes miranda*	31.2 ± 2.6 μg/mL
Leaf extract of *Nepenthes miranda*	71.2 ± 5.0 μg/mL
Leaf extract of *Sinningia bullata*	>1000 μg/mL
Stem extract of *Sinningia bullata*	>1000 μg/mL
Tuber extract of *Sinningia bullata*	N.D.

Values show mean standard deviation of at least three independent experiments.

**Table 3:** Molecular docking analysis against KpSSB.

	S Score	Receptor Residue	Interaction	Distance (Å)	E (kcal/mol)
Plumbagin	−4.1582	Glu101 (C)	H-donor	3.23	−2.0
		Glu101 (C)	H-donor	3.53	−0.5
Sitosterol	−5.8286	Met112 (A)	H-donor	3.75	−0.6
Hydroxyhydroquinone	−3.0747	Glu39 (A)	H-donor	2.87	−2.8
Pyrogallol	−3.3171	Glu51 (A)	H-donor	3.01	−1.2
Hexadecanoic acid	−5.4094	Lys8 (A)	H-acceptor	3.55	−1.2
Oleic acid	−4.3208				
2-Ethyl-3-methylnaphtho[2,3-b]thiophene-4,9-dione	−4.0696	Asn32 (B)	Pi-H	3.82	−0.7
Catechol	−3.4760	Thr53 (A)	H-donor	3.22	−0.8
3-Methoxycatechol	−3.8860				
Methyl α-d-galactopyranoside	−4.0449	Glu51 (B)	H-donor	3.20	−1.9
		Gln117 (B)	H-acceptor	3.04	−1.3

## Data Availability

Not applicable.
